# Mutational Landscape of Virus- and UV-Associated Merkel Cell Carcinoma Cell Lines Is Comparable to Tumor Tissue

**DOI:** 10.3390/cancers13040649

**Published:** 2021-02-05

**Authors:** Kai Horny, Patricia Gerhardt, Angela Hebel-Cherouny, Corinna Wülbeck, Jochen Utikal, Jürgen C. Becker

**Affiliations:** 1 Translational Skin Cancer Research, German Cancer Consortium (DKTK), 45141 Essen, Germany; k.horny@dkfz.de; 2 German Cancer Research Center (DKFZ), 69120 Heidelberg, Germany; 3 Department of Dermatology, University Medicine Essen, 45141 Essen, Germany; patricia.gerhardt@uni-due.de (P.G.); angela.cherouny@uk-essen.de (A.H.-C.); c.wuelbeck@dkfz.de (C.W.); 4 Skin Cancer Unit, German Cancer Research Center (DKFZ), 69120 Heidelberg, Germany; j.utikal@dkfz.de; 5 Department of Dermatology, Venerology and Allergology, University Medical Center Mannheim, 68167 Mannheim, Germany

**Keywords:** merkel cell carcinoma, merkel cell polyoma virus, UV, cell line, MYC, TP53, RB1, whole-exome, significantly mutated genes, copy number variation

## Abstract

**Simple Summary:**

Merkel cell carcinoma (MCC) is an aggressive, rare skin cancer which is caused either by a virus or chronic UV exposure. For both forms, distinct genetic alterations have been described; however, these observations were mostly made in tumor tissue. Since cancer cell lines are frequently used as preclinical models to investigate biological function, we considered it necessary to establish the genomic landscape of MCC cell lines by whole-exome sequencing. We confirmed the presence of UV-induced DNA damage, a high number of mutations and several coding mutations in virus-negative cell lines which were absent in virus-positive cell lines; these, however, harbored characteristic copy number variations, suggesting some virally caused genetic instability. Knowing the genomic features of MCC cell lines validates previous, and facilitates upcoming, experimental studies to discover their biological and translational relevance.

**Abstract:**

Merkel cell carcinoma (MCC) is a rare, highly aggressive cutaneous malignancy that is either associated with the integration of the Merkel cell polyomavirus or chronic UV exposure. These two types of carcinogenesis are reflected in characteristic mutational features present in MCC tumor lesions. However, the genomic characteristics of MCC cell lines used as preclinical models are not well established. Thus, we analyzed the exomes of three virus-negative and six virus-positive MCC cell lines, all showing a classical neuroendocrine growth pattern. Virus-negative cell lines are characterized by a high tumor mutational burden (TMB), UV-light-induced DNA damage, functionally relevant coding mutations, e.g., in *RB1* and *TP53*, and large amounts of copy number variations (CNVs). In contrast, virus-positive cell lines have a low TMB with few coding mutations and lack prominent mutational signatures, but harbor characteristic CNVs. One of the virus-negative cell lines has a local *MYC* amplification associated with high *MYC* mRNA expression. In conclusion, virus-positive and -negative MCC cell lines with a neuroendocrine growth pattern resemble mutational features observed in MCC tissue samples, which strengthens their utility for functional studies.

## 1. Introduction

Merkel cell carcinoma (MCC) is a rare, highly aggressive neuroendocrine skin cancer. It is either associated with chronic Ultraviolet (UV)-light exposure or the genomic integration of the Merkel cell polyomavirus (MCPyV) [1,2]. Virus-associated MCCs are highly prevalent in countries with high latitude, while UV-associated MCCs are more frequent in regions close to the equator [1,2,3].

The different forms of carcinogenesis of MCC are represented in various genomic features, as demonstrated by targeted [4,5,6,7,8,9,10], whole-exome [11,12,13,14] and whole-genome sequencing [15], as well as comparative genomic hybridization (CGH) [16,17,18,19,20,21]. Virus-negative MCCs are characterized by a high tumor mutational burden (TMB), the presence of UV-light-induced DNA damage, functional driver mutations, and high numbers of copy number variations (CNVs). Virus-positive MCCs have a very low TMB and lack known cancer-driving mutations and prominent mutational signatures. Characteristic CNV patterns have repeatedly been reported for virus-associated MCC.

The majority of genomic studies analyzed MCC tissue samples; only a few studies addressed MCC cell lines. CNV patterns of six virus-positive MCC cell lines were previously characterized using CGH [17]. Three virus-negative MCC cell lines, previously characterized by targeted sequencing, have variant growth characteristics [6]. Notably, the origin of “variant” MCC cell lines is controversial, since these have different growth and gene expression patterns to other “classical” virus-negative cell lines [22,23], which share neuroendocrine growth features—i.e., growing in suspension as spheroids—with virus-positive cell lines. Thus, since comprehensive mutational characterization of the MCC cell lines is missing, we analyzed the mutational landscape of cell lines that are frequently used in MCC research by whole-exome sequencing (WES).

## 2. Results

Whole-exome sequencing of the virus-positive cell lines WaGa, MKL-1, UKE-MCC3b, UM-MCC13, UM-MCC29 and PeTa, as well as the virus-negative cell lines UM-MCC9, UM-MCC32 and UM-MCC34, was performed using the SureSelect Exon V6 Kit on a HiSeq 4000 with, on average, 118 million reads per sample. Moreover, we directly compared the cell line PeTa with cryopreserved tissue from which PeTa has been established to assess possible differences between the cell line and tissue.

### 2.1. Mutational Burden and Signatures of MCC Cell Lines Are in Accordance with MCC Tissue Characteristics

**Figure 1:**
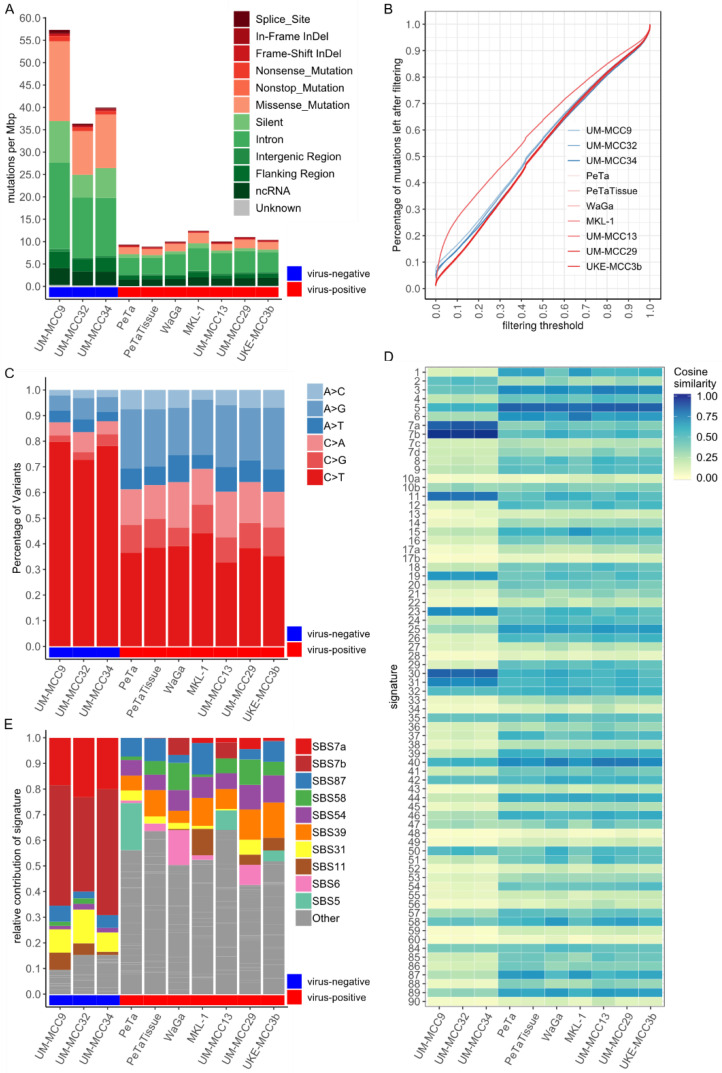
Virus-negative Merkel cell carcinoma (MCC) cell lines show high tumor mutational burden (TMB) and presence of UV-light-induced DNA damage, while virus-positive cell lines have low TMB and lack prominent signatures; (**A**) Mutational burden in mut/Mbp, color-coded by variant classification; (**B**) Filtering of polymorphisms in MCC cell lines showing the relative decrease in TMB (y-axis) with increasing variant allele frequency (VAF) threshold (x-axis) from gnomAD genome database; Figure S1A depicts the same plot with log-transformation of x-axis; (**C**) Contributions of base-pair transitions for single nucleotide variants (SNVs), normalized by total number of SNVs. Complementary transitions are merged in one category (e.g., G > A and C > T as C > T); (**D**) Cosine similarity between trinucleotide context frequencies (TCFs) of MCC cell lines and reference signatures reveals two distinct patterns for virus-positive and -negative cell lines; (**E**) Signature contribution of MCC cell lines after fitting to reference signatures. Signature contributions are normalized to total number of SNVs in the respective cell line. Signatures not reaching at least 10% contribution in at least one sample are summarized as “Other”. Abbreviations: mut/Mbp: Mutations per Megabasepair, SBS: Single Base Substitution.

Virus-negative MCC cell lines have a higher mutational burden with, on average, 44.5 mutations per megabasepairs (mut/Mbp), constituting, on average, 2.693 absolute mutations per cell line than virus-positive MCC cell lines, which contain, on average, 10.5 mut/Mbp (an average of 637 mutations) (Figure 1A, Table S1). Similarly, the number of coding mutations is higher in virus-negative MCC cell lines with, on average, 15.1 mut/Mbp (an average of 913 mutations per cell line, i.e., 33.6% of respective mutations) compared to 2.31 mut/Mbp in virus-positive MCC cell lines (on average, 140 mutations, i.e., 21.9% of mutations). The average fraction of missense (29.3%) and silent (15.6%) mutations in virus-negative cell lines is also higher than in virus-positive cell lines (16.4%/6.8%) (Figure 1A).

Since mutations are called between the respective cell line and the human reference genome hg19, the observed somatic TMB strongly depends on the filtering strategy for potential polymorphisms (Figure 1B and Figure S1). Polymorphisms are identified using the variant allele frequency (VAF) reported in databases covering nonmalignant exomes and genomes. In general, exome databases cover 71.6% of all MCC cell line mutations while genome databases cover either 91.4% in the 1000 genomes database or 97.7% in the “genome aggregation database” (gnomAD) genome database (Figure S1B,C). Therefore, we filtered for VAFs greater than 0.001% with the comprehensive gnomAD genome database. This reduced the presented TMB of virus-positive MCC cell lines by 98.8% from, on average, 54,850 to 637 mutations per cell line. The TMB of virus-negative MCC cell lines shows a smaller reduction by 94.8% from, on average, 52,614, to 913 mutations (Figure 1B and Figure S1A).

Virus-negative MCC cell lines are characterized by a high fraction of, on average, 77% C > T single-nucleotide variations (SNVs), as compared to 38% in virus-positive MCC cell lines (Figure 1C). This observation already suggests different forms of mutagenesis. Hints regarding the underlying mutagenic process can be retrieved from the first preceding and following basepair of an SNV, i.e., the trinucleotide context frequency (TCF). TCFs for virus-negative MCC cell lines show characteristic C > T transition patterns known to be caused by UV-induced mutagenesis (Figure S2) [24]. In contrast, virus-positive MCC cell lines have a “flat” TCF distribution, i.e., low frequencies for most categories, with only slightly elevated C > T and T > C transitions. For MKL-1 and UKE-MCC3b, there is a higher presence of C > T transitions with guanine as the following basepair; a pattern which often originates from spontaneous deamination of CpGs correlating with progressing age. The systematic comparison of the TCFs of MCC cell lines with reference mutational signatures reflecting defined mutagenic processes reveals distinct patterns for virus-negative and -positive cell lines (Figure 1D). Notably, the aging signature 1 and defective DNA mismatch repair signatures 6 and 15 were very similar to the TCF of MKL-1. Fitting reference signatures to the TCFs demonstrates a high contribution of signatures 7a and 7b for virus-negative MCC cell lines (on average, 67.2%), which are both associated with UV-light-induced DNA damage (Figure 1E). Virus-positive MCC cell lines generally have low individual signature contributions, with no prominent mutational signature present: approximately 50% of the total signature contribution for virus-positive MCC cell lines originates from signatures with less than 10% contribution (Figure 1E). Most of the absolute differences in the mutational burden between virus-negative and -positive MCC cell lines are due to signatures 7a and 7b. However, some mutational signatures have slightly higher signature contributions relative to others, namely signature 31 in virus-negative and signatures 5, 6, 11, 39, 54, 58 and 87 in virus-positive MCC cell lines. The reconstruction efficiency after signature fitting is, on average, higher in virus-negative (99.57%) than in virus-positive (96.95%) cell lines.

**Figure 2:**
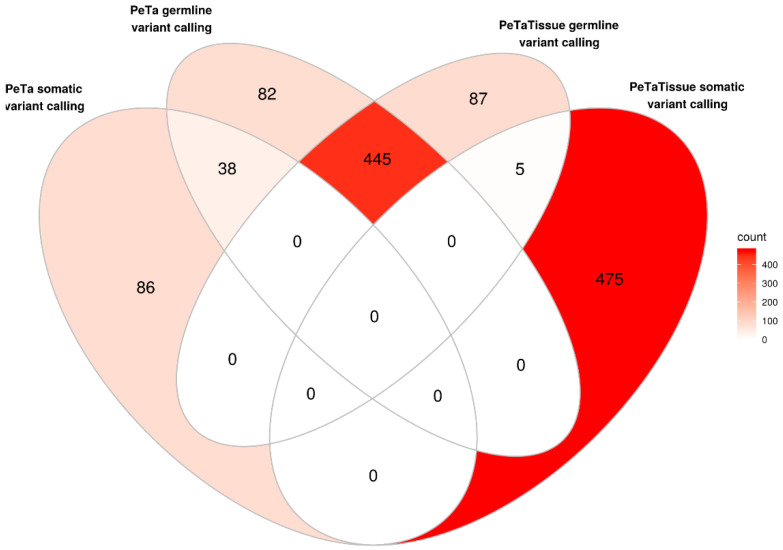
Comparison of unique mutations between PeTa and the respective tumor tissue. Venn-diagram showing how many of the same mutations are shared between the tumor cell line and tissue using either the tissue or the cell line as normal reference for somatic variant calling.

To test if the mutational landscape of the MCC cell lines indeed represents that of the original tumor, we compared the MCC cell line PeTa with cryopreserved tissue from which the cell line was derived (Figure 2, Table S2). The respective exomes share almost 80% of mutations, with 21% (120/565) being unique in the cell line and 17% (92/537) unique in the tumor tissue. Somatic variant calling for the cell line using the tissue as reference retrieved 124 variants, of which 38 (31%) were already among the germline-called variants in PeTa. Vice versa, somatic variant calling for the tissue using the cell line as reference resulted in 480 mutations, of which only five (1%) were present in germline-called variants of the tissue (Figure 2).

### 2.2. Mutations Altering Protein Structure

**Figure 3:**
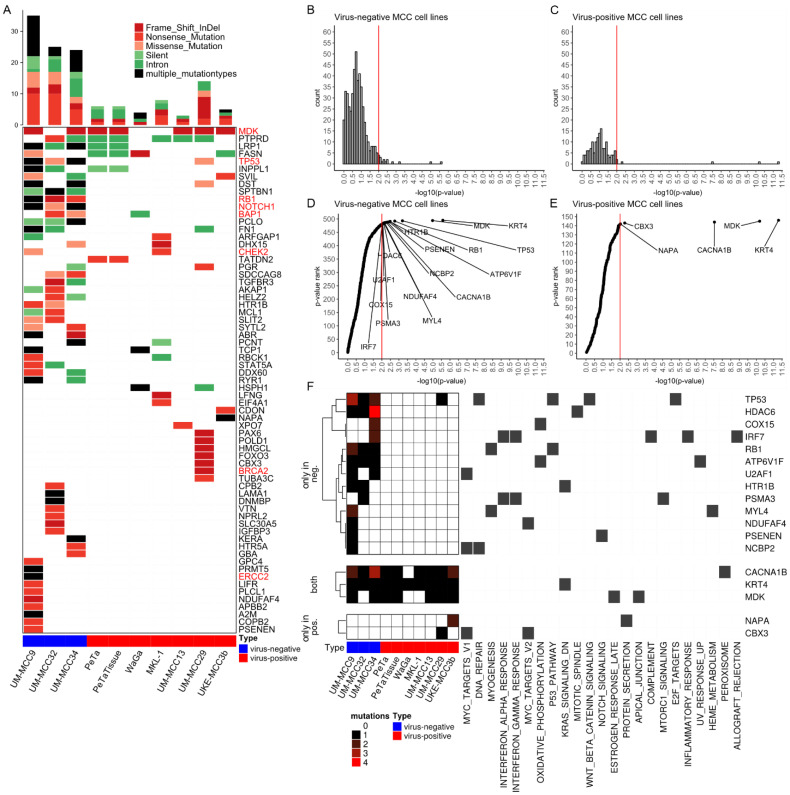
Virus-negative MCC cell lines have high number of coding mutations altering protein structure and significantly mutated genes. (**A**) Oncoplot showing genes selected by the following criteria: (i) containing either a frameshift InDel, nonsense or nonstop mutation and (ii) it is either within a Hallmark Gene Set or its mutation is annotated as pathogenic in ClinVar database. The number of mutations within the selected genes are depicted as bar chart. Both plots are colored by variant classification. Genes emphasized in red are discussed in the results section; (**B**–**E**) Distribution of *p*-values for identification of SMGs as histogram (**B**,**C**) and ranked by *p*-value (**D**,**E**) for virus-negative (**B**,**D**) and -positive (**C**,**E**) MCC cell lines; only genes present in Hallmark Gene Sets were taken into account, red lines indicate a *p*-value of 0.01, genes with a *p*-value of exactly 1 are not shown; (**F**) Mutational burden and involvement in biological processes of SMGs with *p*-value below 0.01 and presence in Hallmark Gene Sets. Abbreviations: MCC: Merkel cell carcinoma, InDel: Insertion and deletion, SMG: Significantly mutated genes.

Next, we investigated mutations predicted to change the amino acid code and likely have an effect on protein function (Figure 3A). Virus-negative MCC cell lines harbor a higher number of nonsense mutations, i.e., mutations introducing a stopcodon, (on average, 53 mutations per cell line, corresponding to 2% of respective mutations) than virus-positive MCC cell lines (on average, six mutations, 0.9% of respective mutations) (Table S3). A total of 21% (≈12 mutations) and 33% (≈2 mutations) of nonsense mutations for virus-negative and -positive cell lines, respectively, are within genes of Hallmark Gene Sets, representing specific biological processes from the molecular signatures database (MSigDB) [25]. Nonsense mutations that are predicted to be pathogenic and cancer-related in ClinVar are in *RB1* in UM-MCC9 (rs794727481) and UM-MCC34 (rs121913304), in *BAP1* in UM-MCC32 (chr3.52437267.G > A), and in the tumor-suppressor gene *CHEK2* in MKL-1 (chr22.29091725.C > T).

Frameshift Insertions and deletions (InDels) are also enriched in absolute numbers in virus-negative MCC cell lines with, on average, 21 mutations (0.8% of respective mutations) compared to 12 mutations (2% of respective mutations) in virus-positive MCC cell lines (Table S3). A total of 18% (≈4 mutations) of virus-negative and 16% (≈2 mutations) of virus-positive frameshift InDels are within Hallmark Gene Sets, among those annotated as pathogenic and cancer-related in ClinVar are *ERCC2* in UM-MCC9 (chr19.45855805.T > -), and *BRCA2* in UM-MCC29 (chr13.32911298.AAAC > -). UM-MCC9 and UM-MCC34 both harbor frameshift deletions in *TP53* (UM-MCC9: chr17.7577070.G > -, UM-MCC34: chr17.7579518.CTTCA > -), and UM-MCC32 a frame-shift deletion in *RB1* (chr13.48919254.CCAGTACCAAAGTTGATAAT > -); the inhibition of both tumor suppressors plays an important role in MCC carcinogenesis [4,5,6,7,8,9,11,12,13,14,26,27,28]. Besides the frameshift Indels and nonsense mutations, there are missense mutations of *TP53* in UM-MCC29 (rs1057520000) and UM-MCC32 (rs121912651). In UM-MCC9, following the frameshift deletion of *TP53,* are a missense (rs786201059) and a silent (chr17.7577558.G > A) mutation, while UM-MCC34 harbors only a silent mutation (chr17.7579516.G > A) before the frameshift deletion. For *RB1*, UM-MCC9 has a missense mutation (rs137853294) following the nonsense mutation, which, therefore, has no effect on the amino acid sequence. There are several other frameshift InDels that likely contribute to MCC carcinogenesis, for example, a frameshift deletion in *NOTCH1* in UM-MCC9 (chr9.139399867.AG > -). Moreover, only two nonstop mutations are found in this study, the first in the transcription repressor *GMNN* [29] in UM-MCC29 (rs757538616) and the other in chaperonin *TCP1* in WaGa (rs779397332) (Table S3).

### 2.3. Significantly Mutated Genes

Next, we tested for genes with a significantly higher mutational burden as expected by chance, aka significantly mutated genes (SMGs) (Figure 3B–F, Tables S4–S6) [30]. In this approach, the mutations of several samples are aggregated and compared with a local background model of silent mutations for each respective gene [30]. This analysis was performed separately for all virus-negative (Figure 3B,D) and for all virus-positive (Figure 3C,E) cell lines. Downstream analysis was restricted to Hallmark Gene sets to focus on genes possibly relevant to MCC carcinogenesis. We evaluated the significance of the respective mutational burden by visualizing the distribution of *p*-values (Figure 3B–E). When correcting all *p*-values for multiple testing using Benjamini–Hochberg procedure, the genes *KRT4*, *MDK* and *CACNA1B* remained the only SMGs in the Hallmark Gene Sets present in both MCC types. Virus-negative MCC cell lines harbor more SMGs with a *p*-value lower than 0.01 within Hallmark Gene Sets compared to virus-positive MCC cell lines, i.e., 16 vs. 5 genes, respectively (Figure 3D–F). Among the SMGs for virus-negative cell lines are *TP53* and *RB1,* which are frequently mutated tumor-suppressor genes in virus-negative MCC (Figure 3F) [4,5,6,7,8,9,11,12,13,14]. Of the SMGs found in virus-positive MCC cell lines, UM-MCC29 has a frame-shift deletion in the chromatin modifier *CBX3* (chr7.26248161.A > -), and UKE-MCC3b a falsely annotated nonstop mutation in *NAPA*, the latter composed of an in-frame insertion (chr19.47998837.- > ATTAAA) and deletion (chr19.47998843.GTT > -), resulting in the addition of two and deletion of one amino acid without introducing a stopcodon (Figure 3F).

We identified three SMGs (*KRT4*, *MDK* and *CACNA1B*) with extraordinary low *p*-values (Figure 3D–F) in all MCC cell lines. *KRT4* contains the exact same large in-frame insertion in all samples (rs11267392), which has a VAF of 87% in the 1000 genomes database. *MDK* comprise the exact same frameshift deletion in a cytosine-rich repeat in six samples, which is actually a mixture of a single-cytosine (chr11.46404342.C > -) and double-cytosine (chr11.46404342.CC > -) deletion. *CACNA1B* has the same large-scale insertion at a splice site in seven samples (chr9.140773612.- > ACGACACGGAGCCCTATTTCATCGGGATCTTTTGCTTCGAGG CAGGGA, rs370237172).

### 2.4. Characteristic Copy Number Variation Patterns in Virus-Positive MCC Cell Lines

**Figure 4:**
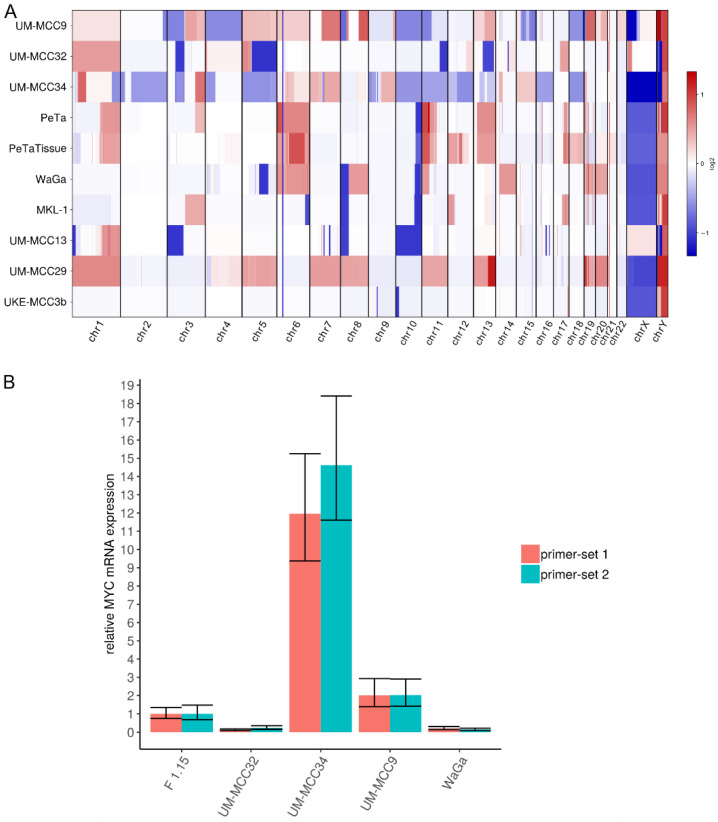
CNVs in MCC cell lines. (**A**) Graphical display of derived CNVs using CNVkit with sex chromosomes relative to haploid reference; (**B**) Expression of *MYC* mRNA in MCC cell lines was determined by qRT-PCR. Cq values were normalized to GAPDH expression and compared to ΔCq value of fibroblasts (F 1.15).

CNVs were determined from the exome sequencing data (Figure 4A, Table S7). The virus-negative cell lines UM-MCC9 and UM-MCC34, but not UM-MCC32, are characterized by numerous, varying CNVs covering most of the genome. Virus-positive MCC cell lines have less, but more characteristic CNVs, which include whole-chromosome gains of chr1 (UM-MCC29), chr5, chr7, chr8 (UM-MCC29), chr6 (WaGa, PeTa), chr11 (UM-MCC29), chr13 (UM-MCC29, PeTa), chr19, chr20 (UM-MCC29, WaGa) and a complete loss of chr10 (UM-MCC13). Several chromosomes are partially amplified, e.g., chr1q (UM-MCC13, PeTa), chr3q (MKL-1, PeTa) and chr11 (WaGa, PeTa), while others show partial losses, such as chr3p (UM-MCC13), chr8p (UM-MCC13, MKL-1, WaGa) and chr10q (MKL-1, WaGa, PeTa). Only UKE-MCC3b lacks any substantial copy number changes.

Previous studies reported copy number losses covering *RB1* on chromosome 13 [4,5,11,12,14,16,31]. We also observe large single-copy deletions on chromosome 13 including the loss of *RB1* in virus-negative cell lines (UM-MCC32 and UM-MCC34); in contrast, there are large single-copy gains that include *RB1* in one virus-negative (UM-MCC9) and two virus-positive cell lines (PeTa, UM-MCC29).

Local amplifications of *MYCL* on chromosome 1 have previously been reported for both MCC types [4,5,16,31]. Here, *MYCL* is included in the whole-chromosome gains of UM-MCC29 and UM-MCC32 as well as the partial chromosome gains in UM-MCC34. Interestingly, UM-MCC34 has an extraordinarily high, localized amplification of *MYC* (aka *c-MYC*), with 106 copies covering ~530,000 basepairs on chromosome 8. *MYC* is also included in larger whole- or partial-chromosome gains in UM-MCC29, WaGa and UM-MCC9. These amplifications are associated with a higher *MYC* mRNA expression, which is most pronounced in UM-MCC34 (Figure 4B).

## 3. Discussion

Due to the lack of suitable genetically engineered mouse models (GEMMs), preclinical functional studies rely on MCC cell lines. However, the detailed genomic characteristics of the applied cell lines are not fully established. Indeed, most studies investigating genomic features of MCC by targeted or WES are based on fresh frozen or formalin-fixed paraffin-embedded (FFPE) tissue samples [4,5,6,7,8,10,11,12,13,14]. Only Wong et al. included three virus-negative cell lines [6] that may be not representative for MCC [22,23]. Here, we present the mutational landscape of three classical virus-negative and six virus-positive MCC cell lines (characteristics are summarized in Table 1). The ratio of virus-positive to -negative cell lines recapitulates the ratio of MCC tumors in countries with high latitude [1]. The genomic features of the MCC cell line cohorts are very similar to those previously reported for the respective MCC tumors. Furthermore, direct comparison of one matched cell line-tissue pair confirmed that genomic alterations accumulated during cell culture only caused minor differences in their mutational landscape. However, expectedly, the cell line did not capture the complete tumor heterogeneity, as many somatic mutations were specific to the tissue.

Virus-positive MCCs are characterized by very low TMB, a lack of prominent mutational signatures and the absence of functional mutations (Table 1) [4,5,6,11,12,13]. Previously reported TMBs for virus-positive MCC, however, show large differences and are inconsistently specified, e.g., regarding normalization. For the WES studies, TMB was reported either as a median of 12.5 SNVs [12], an average of 0.4 mut/Mbp [11] or a median of 1.57 mut/Mbp [13]. We observed, on average, 11 mut/Mbp, which is comparable with studies using targeted sequencing approaches (i.e., an average of 5–10 mut/Mbp [6], a median of 1.2 coding mut/Mbp [5] or up to 16 mut/Mbp [4]). All studies with higher TMB lacked individual normal tissues as a reference for somatic variant calling, hence databases reporting common polymorphisms (e.g., 1000 genomes, exome aggregation consortium (ExAC), gnomAD databases) had to be used for filtering non-somatic variants. Thus, the observed higher TMBs are likely caused by polymorphisms not represented in common databases. This notion is supported by the absence of any prominent mutational signature in virus-positive MCC samples. No single mutational signature has a relevant contribution to the TMB; only “flat” TCF distributions were detected for virus-positive MCC cell lines, which likely represent randomly distributed, unfiltered polymorphisms that may impair the detection of other mutagenic processes. The absence of functional, cancer-related mutations and low signature reconstruction efficiency is in line with this assumption. In contrast, in virus-negative MCC cell lines, TMB is high (on average, 44.5 mut/Mbp), mutational patterns are strongly associated with UV-light-induced DNA damage, and many coding mutations of cancer-related genes exist (Table 1). The primary origin of virus-negative MCC cell lines is associated with UV-exposed areas. UM-MCC9 and UM-MCC32 were derived from primary tumors localized on the scalp, and UM-MCC34 was derived from axillary metastasis presumably originating from a primary tumor on the upper extremity (Table 2) [28]. Some of the virus-positive MCC cell lines were generated from tumors without a clear association with chronic UV-exposure, e.g., PeTa and UKE-MCC3b originated from tumors of the trunk (Table 2) [17]. Interestingly, we did not observe major differences in TMB between cell lines derived from primary tumors (UM-MCC9, UM-MCC32, PeTa) and metastases (UM-MCC34, WaGa, MKL-1, UM-MCC13, UM-MCC29, UKE-MCC3b), which would have been expected from more general observations in cancer (Table 2) [32].

All virus-negative MCC cell lines show *RB1* and *TP53* disruption, either by frameshift deletion, nonsense, missense mutation or, for *RB1,* possibly copy number losses. Alterations in both genes are recurrent mutational features in virus-negative MCC [4,5,6,7,8,9,11,12,13,14,26]. Notably, the exact same nonsense mutations in *RB1* were previously reported for UM-MCC9 (rs794727481 [9,14]) and UM-MCC34 (rs121913304 [4,6]). *RB1* and *TP53* abrogation is also common in other neuroendocrine carcinomas, e.g., in small cell lung, neuroendocrine prostate and pancreatic carcinoma [35]. In this context, it is interesting to note that *MYC* binding motifs are enriched in neuroendocrine genes; thus, it has been proposed that *MYC* overexpression drives the temporal tumor cell evolution [36]. We detected an extraordinarily high *MYC* amplification associated with equally high mRNA expression in UM-MCC34. *MYC* family gene amplification, i.e., 6% for *MYCL* and 4% for *MYC* in virus-negative MCCs [5,16], as well as high *MYC* protein expression, was previously reported [13,37].

The biological importance of SMGs relies on the fact that these may be more prone for mutations due to open chromatin regions, i.e., reflecting the functional state of a cell during mutagenesis, or being positively selected during tumor evolution. The SMGs with extraordinary low *p*-values were *KRT4*, *MDK* and *CACNA1B*, suggesting that these genes may be relevant for MCC carcinogenesis. However, critical examination of these mutations demonstrate that this is very unlikely. The mutations in *KRT4* are present in all cell lines and have been previously identified as a common polymorphism with 87% VAF in the 1000 genomes database. Thus, the *KRT4* mutation is actually the major allele of a single nucleotide polymorphism (SNP) not reflected in the hg19 reference genome. For *MDK*, the detected cytosine deletion is embedded in a sequence of 15 cytosines in close proximity to a stopcodon and is therefore in a region prone to sequencing artifacts. Actually, variations in cytosine counts of this region have already been reported in dbSNP as polymorphisms (rs74916763). Finally, the large-scale insertion in *CACNA1B* is localized at the last basepair of an exon and the inserted sequence is identical to the beginning of the following exon, hence we assume a deletion of an intronic region in between, which has already been reported with 0.3% VAF in the Allele Frequency Aggregator (ALFA) database. Consequently, these three variants likely reflect limitations in the representation and annotation of polymorphisms, which emphasizes the importance of variant filtering and evaluation. In virus-negative MCC cell lines, *TP53* and *RB1* have a relatively low *p*-value compared to other genes (Figure 3D) and, due to their recurrency in MCC, these genes are likely associated with tumorigenesis of virus-negative MCC.

CNVs were previously characterized in MCC using CGH [16,17,18,19,20,21], genome-wide microarrays [31] or next-generation sequencing [4,5,6,7,11,12,15]. We observe higher CNV numbers in virus-negative, and fewer, but characteristic, CNV patterns in virus-positive MCC, indicating a common alteration mechanism for the latter. Notably, the MCPyV-encoded small T antigen was reported to induce centrosome overproduction and to increase the frequency of micronuclei by interaction with E3-ligases, causing chromosome instability [38]. The virus-positive MCC-specific losses and gains may actually affect the tumor suppressor *RB1* and oncogene *MYC*.

*APOBEC*-mediated mutagenesis is a known feature of viral oncogenesis, e.g., in human-papilloma-virus-associated cancer [39]. In our and previously reported studies, *APOBEC* mutations seem to be absent in MCC [4,5]. However, *APOBEC*-related mutagenesis is restricted to localized, hypermutated regions, aka kataegis, that are difficult to detect by WES and even more so by targeted sequencing. Indeed, in whole-genome analysis, an *APOBEC*-related kataegis was reported in a virus-positive MCC [15]. Thus, to detect *APOBEC*-related mutagenesis with enhanced sensitivity, signature analysis should be restricted to such hypermutated regions [40].

In summary, WES of virus-positive and -negative MCC cell lines with a neuroendocrine growth pattern revealed mutational features resembling those previously observed in MCC tissue samples; hence, our report strengthens the utility of these classical MCC cell lines for functional studies.

## 4. Materials and Methods

### 4.1. Cell Lines and Tissues

The MCC cell lines WaGa [33], PeTa [26], MKL-1 [34], UM-MCC13, UM-MCC29, UM-MCC9, UM-MCC32, UM-MCC34 [28] were described before (Table 2). UM-MCC13, UM-MCC29, UM-MCC9, UM-MCC32 and UM-MCC34 were provided by Monique E. Verhaegen, University of Michigan, Ann Arbor, MI, USA. UKE-MCC3b was established at the department of dermatology at the University Medicine Essen, Essen, Germany and the patient gave informed consent (ethics committee approval: 11–4715; 17-7538-BO). WaGa, PeTa, MKL-1, UKE-MCC3b and UM-MCC34 were maintained at 37 °C with 5% CO_2_ in RPMI-1640 with 10% fetal bovine serum (FBS), 1% penicillin/streptomycin (PAN Biotech, Aidenbach, Germany), while UM-MCC13, UM-MCC29 and UM-MCC32 were maintained as described previously [28] in self-renewal media [41] including low-glucose DMEM, Neurobasal-A medium, 2-mercaptoethanol, N-2 Supplement (100× (times)), B-27™ Supplement (50×, minus vitamin A), MEM non-essential amino acids solution (100×), Gibco™ Amphotericin (all Thermo Fischer, Dreieich, Germany), retinoic acid (Sigma Aldrich, Darmstadt, Germany), basic fibroblast growth factor, recombinant human IGF-I (Peprotech, Hamburg, Germany), 1% penicillin/streptomycin (PAN Biotech). The self-renewal medium was further supplemented with chicken embryo extract containing HBSS, PBS (PAN Biotech), MEM with Earle’s salts and L-glutamine (Thermo Fischer) and Hyaluronidase specs (Sigma Aldrich) [42]. Primary cutaneous fibroblasts (F 1.15) were generated and maintained as previously described [43].

### 4.2. Library Preparation and Sequencing

DNA was purified using DNeasy Blood & Tissue Kit (Qiagen, Hilden, Germany). Library preparation and sequencing were performed by DKFZ Genomics and Proteomics Core Facility. WES libraries were prepared using SureSelect All Exon V6 Kit (Agilent Technologies, Santa Clara, CA, USA) and subsequently sequenced on HiSeq 4000 (Illumina) paired-end 100bp reads with, on average, 118 million reads per sample.

### 4.3. Alignment and Variant Calling

Processing of reads in FASTQ format to genomic variations in variant call format (VCF) was performed according to genome analysis toolkit (GATK) best practices of germline short variant discovery for all MCC cell lines. Additionally, for PeTa and PetaTissue, GATK best practices of somatic short variant discovery were used. Paired-end reads in FASTQ Format were aligned to the human reference genome hg19 (GRCh37) using Burrows–Wheeler aligner (BWA) mem v0.7.17 [44]; duplicates were marked using Picard MarkDuplicates and aligned reads sorted using samtools v1.7. GATK Toolkits of version 4.0.12.0 were used. For germline short variant discovery, GATK BaseRecalibrator and ApplyBQSR were applied and, subsequently, variants were called using GATK HaploTypeCaller without normal tissue reference data. For somatic short-variant discovery, the panel of normal (PoN) for PeTa and PeTaTissue were created and variants were called with GATK Mutect2, once with Peta cell line and once with PeTaTissue as normal reference. Variants were annotated using ANNOVAR (Version from 8 June 2020) and databases of Ensembl Gencode v31 (29 September 2019), dbSNP with allelic splitting and left-normalization v150 (29 September 2017) ClinVar (05 March 2015), ExAC (29 November 2015), gnomAD exome and genome collection (v2.1.1, 18 March 2019), 1000 genomes dataset (24 August 2015) and Kaviar database (03 December 2015) were used.

### 4.4. Variant Filtering

The Maftools R package v2.0.05 was used for VCF to Mutation Annotation Format (MAF) conversion using ensemble genes as gene column, and used for manipulation of MAF files in R [45]. Variants that are not within the probe region of the SureSelect All Exon V6 Kit were removed from analysis. Variants from germline variant calling were filtered and removed from analysis if one of the following criteria was met: SNVs with QD < 2.0, MQ < 50.0, FS > 60.0, SOR > 5.0, MQRankSum < −12.5 or ReadPosRankSum < −8.0 and InDels with QD < 2.0, FS > 200.0, SOR > 10.0, InbreedingCoeff < −0.8 or ReadPosRankSum < −20.0. For evaluation of subsequent filtering of possible polymorphisms, we compared different databases reporting VAFs (Figure S1B,C). Based on this analysis, we filtered a variant as germline polymorphism if it reported a VAF of more than 0.001% in gnomAD v2.1.1 genome. Variants from somatic variant calling of Peta/PeTaTissue were filtered using GATK FilterMutectCalls and not filtered for germline polymorphisms.

### 4.5. Quantitative Real-Time PCR (qRT-PCR)

RNA of cell lines was extracted using peqlabGold Micro RNA Kit (PEQLAB Biotechnologie GmbH, Erlangen, Germany) and transcribed to cDNA using SuperScript IV Reverse Transcriptase (1000u, Life Technologies GmbH, Darmstadt, Germany). qRT-PCR was performed on the CFX Real-Time PCR system (Bio-Rad Laboratories, Hercules, CA, USA) using LuminoCT SYBR Green qPCR ready Mix (Sigma Aldrich). For *MYC* following primers were used: primer-set 1 forward: GGCTCCTGGCAAAAGGTCA, reverse: CTGCGTAGTTGTGCTGATGT; primer-set 2 forward: GTCAAGAGGCGAACACACAAC, reverse: TTGGACGGACAGGATGTATGC. *GAPDH* primer set: forward ACCACAGTCCATGCCATCAC, reverse TCCACCACCCTGTTGCTGTA. Annealing was performed at 60 °C for 15 s. Relative quantification was performed using the 2^−ΔΔCq^ method implemented in the R package “pcr” [46].

### 4.6. Bioinformatic Processing

The R Markdown script for analysis of MAF format files is attached in File S1. Normalization to mutations per Megabasepair was done through dividing the number of mutations by the sum of the length of all regions covered by probes (60,456,963 basepairs). Signature analysis was performed using only SNVs and MutationalPatterns R package v1.8.0 [47]. Reference mutational signatures of version 3.1 [48] were downloaded from the Cataloque of Somatic Mutations in Cancer (COSMIC, https://cancer.sanger.ac.uk/cosmic/signatures, (accessed date 1 October 2020)). SMGs were determined using MutSigCV v1.41 [30] and Hallmark Gene Sets v7.1 downloaded from MSigDB was used [25]. Heatmaps were created using ComplexHeatmap R package v2.1.0 [49], other plots with ggplot v3.1.0 [50] and ggVennDiagram using R programming language v3.5.2. CNVs were derived using CNVkit v0.9.6 with default settings.

## 5. Conclusions

Virus-negative MCC cell lines show high TMB, UV-light DNA damage and several functional coding mutations, while virus-positive MCC cell lines harbor few mutations. Thus, the mutational landscape of MCC cell lines that are frequently used in preclinical research reflect the observations from tumor tissue and confirm their suitability for functional studies.

## Figures and Tables

**Table 1:** Genomic features of MCC cell lines for both MCC types.

MCC Cell Line Type	Virus-Negative	Virus-Positive
**Tumor Mutational Burden**	high (on average, 44.5 mut/Mbp)	low (on average, 10.5 mut/Mbp)
**Mutagenic processes detected**	UV-light-associated DNA damage (SBS7a, SBS7b)	flat mutation profile without prominent signatures.
**Coding mutations**	many mutations with potential functional effect; many mutated genes	few mutations with potential functional effect; few mutated genes
**Copy Number** **Variations**	many widespread CNVs	few, characteristic CNVs

The bold is used to emphasize the row names for the subsequent summary.

**Table 2:** Overview of analyzed Merkel cell carcinoma cell lines.

Cell Line	MCPyV Status	Established From	Localization of Primary	Time in Culture	Reference
UM-MCC9	negative	primary, scalp	scalp	>6 years	[28]
UM-MCC32	negative	primary, scalp	scalp	>6 years	[28]
UM-MCC34	negative	axillary lymph node metastasis	presumably arm	>6 years	[28]
PeTa	positive	primary, trunk	trunk	>7 years	[17,26]
WaGa	positive	malignant ascites	head	>10 years	[17,33]
MKL-1	positive	nodal metastasis	unknown	>30 years	[17,34]
UM-MCC13	positive	metastasis, leg	presumably leg	>6 years	[28]
UM-MCC29	positive	inguinal lymph node metastasis	presumably leg	>6 years	[28]
UKE-MCC3b	positive	metastasis, trunk	trunk	>3 years	-

## Data Availability

The data presented in this study is available in Tables S1 and S2. FASTQ Files are available on request from the corresponding author. The FASTQ Files are not publicly available due to privacy reasons.

## Supplementary Materials

The following are available online at https://www.mdpi.com/2072-6694/13/4/649/s1, Figure S1: Filtering of polymorphisms in MCC cell lines, Figure S2: TCF for each MCC cell line, Table S1: Mutations found in MCC cell lines, Table S2: Mutations from somatic variant calling of PeTa and tissue of PeTa, Table S3: All nonsense, frameshift and nonstop mutations found in MCC cell lines with respective Hallmark Gene Set, Table S4: Mutations found in SMGs with *p* < 0.01 and within a Hallmark Gene Set, Table S5: SMGs from MutSigCV of virus-negative MCC cell lines, Table S6: SMGs from MutSigCV of virus-positive MCC cell lines, Table S7: CNVs found in MCC cell lines, File S1: R Markdown script used for analysis of MAF files.

## References

[1] Becker J.C., Stang A., DeCaprio J.A., Cerroni L., Lebbé C., Veness M., Nghiem P. (2017). Merkel cell carcinoma. Nat. Rev. Dis. Primers.

[2] Feng H., Shuda M., Chang Y., Moore P.S. (2008). Clonal Integration of a Polyomavirus in Human Merkel Cell Carcinoma. Science.

[3] Garneski K.M., Warcola A.H., Feng Q., Kiviat N.B., Helen Leonard J., Nghiem P. (2009). Merkel Cell Polyomavirus Is More Frequently Present in North American than Australian Merkel Cell Carcinoma Tumors. J. Investig. Dermatol..

[4] Starrett G.J., Thakuria M., Chen T., Marcelus C., Cheng J., Nomburg J., Thorner A.R., Slevin M.K., Powers W., Burns R.T. (2020). Clinical and molecular characterization of virus-positive and virus-negative Merkel cell carcinoma. Genome Med..

[5] Knepper T.C., Montesion M., Russell J.S., Sokol E.S., Frampton G.M., Miller V.A., Albacker L.A., McLeod H.L., Eroglu Z., Khushalani N.I. (2019). The Genomic Landscape of Merkel Cell Carcinoma and Clinicogenomic Biomarkers of Response to Immune Checkpoint Inhibitor Therapy. Clin. Cancer Res..

[6] Wong S.Q., Waldeck K., Vergara I.A., Schröder J., Madore J., Wilmott J.S., Colebatch A.J., De Paoli-Iseppi R., Li J., Lupat R. (2015). UV-Associated Mutations Underlie the Etiology of MCV-Negative Merkel Cell Carcinomas. Cancer Res..

[7] Harms P.W., Collie A.M.B., Hovelson D.H., Cani A.K., Verhaegen M.E., Patel R.M., Fullen D.R., Omata K., Dlugosz A.A., Tomlins S.A. (2016). Next generation sequencing of Cytokeratin 20-negative Merkel cell carcinoma reveals ultraviolet-signature mutations and recurrent TP53 and RB1 inactivation. Mod. Pathol..

[8] Veija T., Sarhadi V.K., Koljonen V., Bohling T., Knuutila S. (2016). Hotspot mutations in polyomavirus positive and negative Merkel cell carcinomas. Cancer Genet..

[9] Cohen P.R., Tomson B.N., Elkin S.K., Marchlik E., Carter J.L., Kurzrock R. (2016). Genomic portfolio of Merkel cell carcinoma as determined by comprehensive genomic profiling: Implications for targeted therapeutics. Oncotarget.

[10] Graves C.A., Jones A., Reynolds J., Stuart J., Pirisi L., Botrous P., Wells J. (2015). Neuroendocrine Merkel Cell Carcinoma Is Associated with Mutations in Key DNA Repair, Epigenetic and Apoptosis Pathways: A Case-Based Study Using Targeted Massively Parallel Sequencing. Neuroendocrinology.

[11] Harms P.W., Vats P., Verhaegen M.E., Robinson D.R., Wu Y.-M., Dhanasekaran S.M., Palanisamy N., Siddiqui J., Cao X., Su F. (2015). The Distinctive Mutational Spectra of Polyomavirus-Negative Merkel Cell Carcinoma. Cancer Res..

[12] Goh G., Walradt T., Markarov V., Blom A., Riaz N., Doumani R., Stafstrom K., Moshiri A., Yelistratova L., Levinsohn J. (2015). Mutational landscape of MCPyV-positive and MCPyV-negative Merkel cell carcinomas with implications for immunotherapy. Oncotarget.

[13] González-Vela M.d.C., Curiel-Olmo S., Derdak S., Beltran S., Santibañez M., Martínez N., Castillo-Trujillo A., Gut M., Sánchez-Pacheco R., Almaraz C. (2017). Shared Oncogenic Pathways Implicated in Both Virus-Positive and UV-Induced Merkel Cell Carcinomas. J. Investig. Dermatol..

[14] Cimino P.J., Robirds D.H., Tripp S.R., Pfeifer J.D., Abel H.J., Duncavage E.J. (2014). Retinoblastoma gene mutations detected by whole exome sequencing of Merkel cell carcinoma. Mod. Pathol..

[15] Starrett G.J., Marcelus C., Cantalupo P.G., Katz J.P., Cheng J., Akagi K., Thakuria M., Rabinowits G., Wang L.C., Symer D.E. (2017). Merkel Cell Polyomavirus Exhibits Dominant Control of the Tumor Genome and Transcriptome in Virus-Associated Merkel Cell Carcinoma. mBio.

[16] Paulson K.G., Lemos B.D., Feng B., Jaimes N., Peñas P.F., Bi X., Maher E., Cohen L., Helen Leonard J., Granter S.R. (2009). Array-CGH Reveals Recurrent Genomic Changes in Merkel Cell Carcinoma Including Amplification of L-Myc. J. Investig. Dermatol..

[17] Schrama D., Sarosi E.-M., Adam C., Ritter C., Kaemmerer U., Klopocki E., König E.-M., Utikal J., Becker J.C., Houben R. (2019). Characterization of six Merkel cell polyomavirus-positive Merkel cell carcinoma cell lines: Integration pattern suggest that large T antigen truncating events occur before or during integration. Int. J. Cancer.

[18] Van Gele M., Leonard J.H., Van Roy N., Van Limbergen H., Van Belle S., Cocquyt V., Salwen H., De Paepe A., Speleman F. (2002). Combined karyotyping, CGH and M-FISH analysis allows detailed characterization of unidentified chromosomal rearrangements in Merkel cell carcinoma. Int. J. Cancer.

[19] Van Gele M., Speleman F., Vandesompele J., Van Roy N., Leonard J.H. (1998). Characteristic pattern of chromosomal gains and losses in Merkel cell carcinoma detected by comparative genomic hybridization. Cancer Res..

[20] Larramendy M.L., Koljonen V., Böhling T., Tukiainen E., Knuutila S. (2004). Recurrent DNA copy number changes revealed by comparative genomic hybridization in primary Merkel cell carcinomas. Mod. Pathol..

[21] Härle M., Arens N., Moll I., Back W., Schulz T., Scherthan H. (1996). Comparative genomic hybridization (CGH) discloses chromosomal and subchromosomal copy number changes in Merkel cell carcinomas. J. Cutan. Pathol..

[22] Gele M.V., Boyle G.M., Cook A.L., Vandesompele J., Boonefaes T., Rottiers P., Roy N.V., De Paepe A., Parsons P.G., Leonard J.H. (2004). Gene-expression profiling reveals distinct expression patterns for Classic versus Variant Merkel cell phenotypes and new classifier genes to distinguish Merkel cell from small-cell lung carcinoma. Oncogene.

[23] Daily K., Coxon A., Williams J.S., Lee C.-C.R., Coit D.G., Busam K.J., Brownell I. (2015). Assessment of Cancer Cell Line Representativeness Using Microarrays for Merkel Cell Carcinoma. J. Investig. Dermatol..

[24] Alexandrov L.B., Nik-Zainal S., Wedge D.C., Aparicio S.A.J.R., Behjati S., Biankin A.V., Bignell G.R., Bolli N., Borg A., Børresen-Dale A.-L. (2013). Signatures of mutational processes in human cancer. Nature.

[25] Liberzon A., Birger C., Thorvaldsdóttir H., Ghandi M., Mesirov J.P., Tamayo P. (2015). The Molecular Signatures Database (MSigDB) hallmark gene set collection. Cell Syst..

[26] Houben R., Dreher C., Angermeyer S., Borst A., Utikal J., Haferkamp S., Peitsch W.K., Schrama D., Hesbacher S. (2013). Mechanisms of p53 restriction in Merkel cell carcinoma cells are independent of the Merkel cell polyoma virus T antigens. J. Investig. Dermatol..

[27] Hesbacher S., Pfitzer L., Wiedorfer K., Angermeyer S., Borst A., Haferkamp S., Scholz C.-J., Wobser M., Schrama D., Houben R. (2016). RB1 is the crucial target of the Merkel cell polyomavirus Large T antigen in Merkel cell carcinoma cells. Oncotarget.

[28] Verhaegen M.E., Mangelberger D., Weick J.W., Vozheiko T.D., Harms P.W., Nash K.T., Quintana E., Baciu P., Johnson T.M., Bichakjian C.K. (2014). Merkel cell carcinoma dependence on bcl-2 family members for survival. J. Investig. Dermatol..

[29] Dhar S.K., Yoshida K., Machida Y., Khaira P., Chaudhuri B., Wohlschlegel J.A., Leffak M., Yates J., Dutta A. (2001). Replication from oriP of Epstein-Barr Virus Requires Human ORC and Is Inhibited by Geminin. Cell.

[30] Lawrence M.S., Stojanov P., Polak P., Kryukov G.V., Cibulskis K., Sivachenko A., Carter S.L., Stewart C., Mermel C.H., Roberts S.A. (2013). Mutational heterogeneity in cancer and the search for new cancer-associated genes. Nature.

[31] Carter M.D., Gaston D., Huang W.-Y., Greer W.L., Pasternak S., Ly T.Y., Walsh N.M. (2018). Genetic profiles of different subsets of Merkel cell carcinoma show links between combined and pure MCPyV-negative tumors. Hum. Pathol..

[32] Patel S.A., Rodrigues P., Wesolowski L., Vanharanta S. (2021). Genomic control of metastasis. Br. J. Cancer.

[33] Houben R., Shuda M., Weinkam R., Schrama D., Feng H., Chang Y., Moore P.S., Becker J.C. (2010). Merkel cell polyomavirus-infected Merkel cell carcinoma cells require expression of viral T antigens. J. Virol..

[34] Rosen S.T., Gould V.E., Salwen H.R., Herst C.V., Le Beau M.M., Lee I., Bauer K., Marder R.J., Andersen R., Kies M.S. (1987). Establishment and characterization of a neuroendocrine skin carcinoma cell line. Lab. Investig..

[35] Rickman D.S., Beltran H., Demichelis F., Rubin M.A. (2017). Biology and evolution of poorly differentiated neuroendocrine tumors. Nat. Med..

[36] Ireland A.S., Micinski A.M., Kastner D.W., Guo B., Wait S.J., Spainhower K.B., Conley C.C., Chen O.S., Guthrie M.R., Soltero D. (2020). MYC Drives Temporal Evolution of Small Cell Lung Cancer Subtypes by Reprogramming Neuroendocrine Fate. Cancer Cell.

[37] Shao Q., Kannan A., Lin Z., Stack B.C., Suen J.Y., Gao L. (2014). BET Protein Inhibitor JQ1 Attenuates Myc-Amplified MCC Tumor Growth In Vivo. Cancer Res..

[38] Kwun H.J., Wendzicki J.A., Shuda Y., Moore P.S., Chang Y. (2017). Merkel cell polyomavirus small T antigen induces genome instability by E3 ubiquitin ligase targeting. Oncogene.

[39] Burns M.B., Temiz N.A., Harris R.S. (2013). Evidence for APOBEC3B mutagenesis in multiple human cancers. Nat. Genet..

[40] Maura F., Degasperi A., Nadeu F., Leongamornlert D., Davies H., Moore L., Royo R., Ziccheddu B., Puente X.S., Avet-Loiseau H. (2019). A practical guide for mutational signature analysis in hematological malignancies. Nat. Commun..

[41] Molofsky A.V., He S., Bydon M., Morrison S.J., Pardal R. (2005). Bmi-1 promotes neural stem cell self-renewal and neural development but not mouse growth and survival by repressing the p16Ink4a and p19Arf senescence pathways. Genes Dev..

[42] Stemple D.L., Anderson D.J. (1992). Isolation of a stem cell for neurons and glia from the mammalian neural crest. Cell.

[43] Fan K., Spassova I., Gravemeyer J., Ritter C., Horny K., Lange A., Gambichler T., Ødum N., Schrama D., Schadendorf D. (2020). Merkel cell carcinoma-derived exosome-shuttle miR-375 induces fibroblast polarization by inhibition of RBPJ and p53. Oncogene.

[44] Li H., Durbin R. (2009). Fast and accurate short read alignment with Burrows-Wheeler transform. Bioinformatics.

[45] Mayakonda A., Lin D.C., Assenov Y., Plass C., Koeffler H.P. (2018). Maftools: Efficient and comprehensive analysis of somatic variants in cancer. Genome Res..

[46] Ahmed M., Kim D.R. (2018). pcr: An R package for quality assessment, analysis and testing of qPCR data. PeerJ.

[47] Blokzijl F., Janssen R., van Boxtel R., Cuppen E. (2018). MutationalPatterns: Comprehensive genome-wide analysis of mutational processes. Genome Med..

[48] Alexandrov L.B., Kim J., Haradhvala N.J., Huang M.N., Tian Ng A.W., Wu Y., Boot A., Covington K.R., Gordenin D.A., Bergstrom E.N. (2020). The repertoire of mutational signatures in human cancer. Nature.

[49] Gu Z., Eils R., Schlesner M. (2016). Complex heatmaps reveal patterns and correlations in multidimensional genomic data. Bioinformatics.

[50] Wickham H. (2016). Ggplot2: Elegant Graphics for Data Analysis.

