# Supplementary material for: Mutational Landscape of Virus- and UV-Associated Merkel Cell Carcinoma Cell Lines Is Comparable to Tumor Tissue
Source: Cancers (Basel). 2021 Feb 5;13(4):649. doi: 10.3390/cancers13040649 (PMC7914758; doi:10.3390/cancers13040649)
Supplement: Supplementary file 1 [file cancers-13-00649-s001.zip › Supplementary Figures.docx]

Supplementary Material: Mutational Landscape of Virus- and UV-Associated Merkel Cell Carcinoma Cell Lines Is Comparable to Tumor Tissue

Kai Horny, Patricia Gerhardt, Angela Hebel-Cherouny, Corinna Wülbeck, Jochen Utikal and Jürgen C. Becker


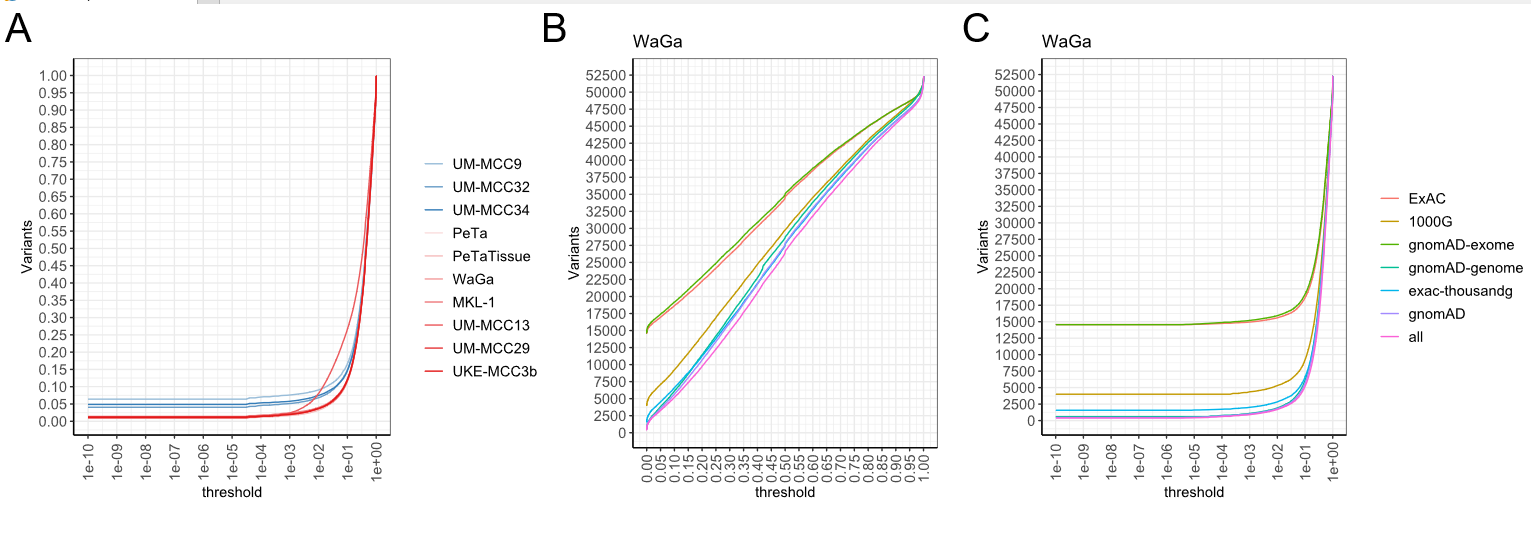


**Figure S1.** Filtering of polymorphisms in MCC cell lines.


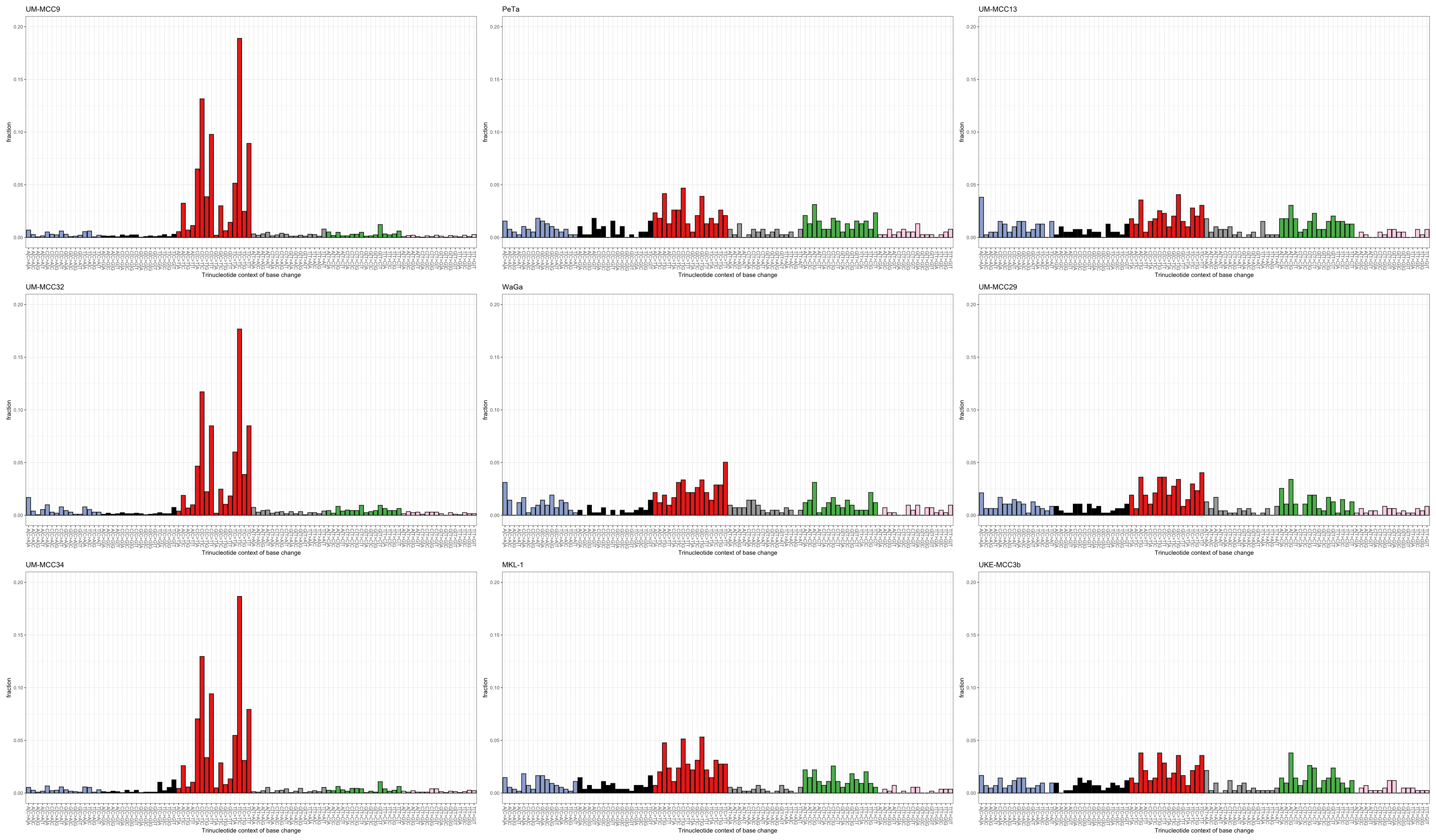


**Figure S2.** TCFs for each MCC cell line.

**Table S1 (available in separate CSV file).** Mutations found in MCC cell lines.

**Table S2 (available in separate CSV file).** Mutations from somatic variant calling of PeTa and tissue of PeTa.

**Table S3 (available in separate CSV file).** All nonsense, frameshift and nonstop mutations found in MCC cell lines with respective Hallmark Gene Set.

**Table S4 (available in separate CSV file).** Mutations found in SMGs with *p* < 0.01 and within a Hallmark Gene Set.

**Table S5 (available in separate CSV file).** SMGs from MutSigCV of virus-negative MCC cell lines.

**Table S6 (available in separate CSV file).** SMGs from MutSigCV of virus-positive MCC cell lines.

**Table S7 (available in separate CSV file).** CNVs found in MCC cell lines, File S1: R Markdown script used for analysis of MAF files.

**File S1 (available in separate R Markdown file).** R Markdown script used for analysis of MAF files.

**Publisher’s Note:** MDPI stays neutral with regard to jurisdictional claims in published maps and institutional affiliations.

| 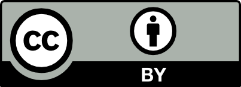 | © 2021 by the authors. Licensee MDPI, Basel, Switzerland. This article is an open access article distributed under the terms and conditions of the Creative Commons Attribution (CC BY) license (http://creativecommons.org/licenses/by/4.0/). |
| --- | --- |
